# Great Effects and Little Causes

**Published:** 1849-07

**Authors:** 


### GREAT EFFECTS AND LITTLE CAUSES.

The same connection between small things and great, runs through all the concerns of our world. The ignorance of a physician, or the carelessness ot an apothecary, may spread death through a family or town. How often has the sickness of one man become the sickness of thousands! How often has the error of one man become the error of thousands! A fly on an atom may set in motion a train of intermediate causes, which shall produce a revolution in a kingdom. Any one of a thousand incidents might have cut off Alexander of Greece in his cradle. But if Alexander had died in his infancy, or had lived a single day longer than he did, it might have put a different face on all the following history of the world. A spectacle maker's boy, amusing himself in his father's shop, by holding two glasses between his finger and his thumb, and varying their distance, perceived the weathercock of the church spire, opposite him, much larger than ordinary, and apparently much nearer, and turned upside down. This excited the wonder of his father, and led him to additional experiments, and these resulted in that astonishing instrument, the telescope, as invented by Galileo, and perfected by Herschel.

On the same optical principle was constructed the microscope, by which we perceive that a drop of stagnate water is a world teeming with inhabitants. By one of these instruments, the experimental philosopher measured the ponderous globes that the Omnipotent hand has ranged in majestic order through the skies; by the other he sees the same hand employed in rounding and polishing five thousand minute transparent globes in the eye of the fly. Yet all these discoveries of modern sciences, exhibiting the intelligence of God, we owe to the transient amusement of a child. It is a fact commonly known, that the laws of gravitation, which guide the thousands of rolling worlds in the planetary system, were suggested at first to the mind of Newton, by the falling of an apple.

The art of printing shows from what casual incidents the most magnificent events in the schemes of providence may result. Time was, when princes were scarcely rich enough to purchase a copy of the Bible. Now, every cottager in Christendom is rich enough to possess this treasure. Who would have thought that the simple circumstance of a man amusing himself by cutting a few letters on the bark of a tree, and impressing them on paper, was intimately connected with the mental illumination of the world.—[Lou. Ed.

